# Preparation of Nanoemulsions with Low-Molecular-Weight Collagen Peptides from Sturgeon Fish Skin and Evaluation of Anti-Diabetic and Wound-Healing Effects in Mice

**DOI:** 10.3390/pharmaceutics15092304

**Published:** 2023-09-12

**Authors:** Nian-Ting Hou, Bing-Huei Chen

**Affiliations:** 1 Department of Food Science, Fu Jen Catholic University, New Taipei City 242062, Taiwan; sudden1996life@gmail.com; 2 Department of Nutrition, China Medical University, Taichung 40402, Taiwan

**Keywords:** sturgeon fish skin, collagen peptide, peptide nanoemulsion, mice, diabetes, wound healing

## Abstract

This study aims to isolate collagen peptides from waste sturgeon fish skin, and prepare nanoemulsions for studying their anti-diabetic and wound-healing effects in mice. Collagen peptides were extracted and purified by acetic acid with sonication, followed by two-stage hydrolysis with 0.1% pepsin and 5% flavourzyme, and ultrafiltration with 500 Da molecular weight (MW) cut-off dialysis membrane. Animal experiments were performed with collagen peptides obtained by pepsin hydrolysis (37 kDa) and pepsin plus flavourzyme hydrolysis (728 Da) as well as their nanoemulsions prepared at two different doses (100 and 300 mg/kg/day). The mean particle size of low-MW and low-dose nanoemulsion, low-MW and high-dose nanoemulsion, high-MW and low-dose nanoemulsion and high-MW and high-dose nanoemulsion was, respectively, 16.9, 15.3, 28.1 and 24.2 nm, the polydispersity index was 0.198, 0.215, 0.231 and 0.222 and zeta potential was −61.2, −63.0, −41.4 and −42.7 mV. These nanoemulsions were highly stable over a 90-day storage period (4 °C and 25 °C) and heating at 40–100 °C (0.5–2 h). Experiments in mice revealed that the low-MW and high-dose nanoemulsion was the most effective in decreasing fasting blood glucose (46.75%) and increasing wound-healing area (95.53%). Collectively, the sturgeon fish skin collagen peptide-based nanoemulsion is promising for development into a health food or wound-healing drug.

## 1. Introduction

Danube sturgeon fish (*Acipenser baerii* × Huso huso), a migratory species of large fish, usually take about 8–10 years to mature through artificial breeding. Because of its meat tenderness without fishy smell and fish bones, sturgeon fish is a popular gourmet meat in Taiwan. However, during the processing of sturgeon fish, a large amount of waste occurs, including fish skin (1.1%), fish bone (0.7%), fish oil (3.4%), fish tail (3.0%) and fish scale (2.9%), in which fish skin, fish bone and fish scale are rich in collagen [1]. Despite a higher level of collagen in fish scales, fish skin is usually used as the raw material for collagen extraction as it would be difficult to extract collagen from fish scales which are tightly combined with outer shells [2]. 

Accordingly, there are three types of collagen with type I accounting for 90% and mainly present in skin, bones, blood vessels, organs and cartilage, while type II collagen is mainly present in joints, cornea and lens and type III collagen in blood vessels and viscera [3]. It has been well documented that collagen, a vital protein mainly present in connective tissue in human body, possesses many important physiological functions such as maintenance of skin elasticity and high stretching ability as well as for wound dressings and tissue structure [2]. However, collagen can be hydrolyzed into peptide or amino acid using enzymes in vivo. Nevertheless, bioactive peptides, composed of short length amino acids (3 to 20), have been shown to possess vital biological activities such as anti-oxidation, anti-microbial infection, anti-hypertension, anti-diabetic and anti-cancer [3].

Diabetes, a metabolic disorder caused by elevated levels of blood glucose in the human body, can be divided into two types: type I and type II, with the former being an insulin-dependent diabetes caused by dysfunction and destruction of pancreatic β-cells leading to deficiency in insulin secretion, and the latter due to excessive insulin secretion resulting in the malfunction of insulin receptors so that insulin resistance occurs, accompanied by hyperinsulinemia symptom [3]. Currently, type I diabetes accounts for about 10% of total diabetes cases, while type II diabetes accounts for 90%, with the latter being closely associated with lifestyle and eating habits. The clinical treatments with drugs in reducing blood glucose level or insulin resistance are often accompanied by side effects. Thus, it is urgent to develop a botanic drug without side effects. The potential of using bioactive peptides in the treatment of diabetes has been reported by several authors. For instance, Rivero-Pino et al. [4] found that the peptide with MW < 1400 Da, isolated from Sardine pilchardus with enzymes including subtilisin, trypsin and flavourzyme followed by purification with SEC, was effective in improving diabetes through the binding of the DPP-IV inhibitor. In another study, a reduction in blood glucose level (35–37%) in mice after oral administration with 50 mg/kg of protein hydrolysate obtained from *Palmaria palmata* for 18 days was observed by McLaughlin et al. [5].

Furthermore, one of the most important complications for diabetic patients is the loss of the skin wound-healing mechanism leading to the occurrence of diabetic foot ulcers. The cellular events involved in four phases of diabetic wound healing are as follows [6]: 1. Hemostasis: vasoconstriction and blood clotting occurs to prevent excess loss of blood and thereby provides a structural matrix for cell migration after injury. 2. Inflammation: neutrophils release ROS and proteases to prevent contamination of bacteria and clean the wound after reaching the wound site. 3. Proliferation: formation of granulation tissue, new blood vessels and epithelialization. 4. Remodeling: myofibroblasts, macrophages and endothelial cells undergo apoptosis and type III collagen is replaced by type I collagen for its tensile strength and wound contraction.

Several studies have demonstrated that collagen peptides are effective in improving diabetes and wound healing in rats and mice [7,8]. However, collagen peptides may undergo hydrolysis via acid and enzymes in the gastrointestinal tract in vivo. To overcome this issue, the development of an appropriate nanosystem for encapsulation of bioactive peptides is extremely important. For instance, silver nanoparticles were reported to be effective in diabetic wound therapy [9]. Thus, it is feasible to develop a nanoparticle delivery system for the encapsulation of bioactive peptides for treatment of diabetes and wound healing.

The objective of this study was to extract and purify collagen peptides from sturgeon fish skin for subsequent preparation of a nanoemulsion system for encapsulation of bioactive peptides to improve diabetes and wound healing in mice.

## 2. Materials and Methods

### 2.1. Materials 

A total of 1 kg sturgeon fish (*Acipenser baerii* × Huso huso) skin was provided by Huang-Tsan Co. (New Taipei City, Taiwan) and was freeze-dried to obtain about 400 g for storage at −30 °C for subsequent experiments. Flavourzyme was procured from Sigma-Aldrich Co. (St. Louis, MO, USA). Deionized water was made using a Milli-Q water purification system (Millipore Co., Bedford, MA, USA). Reagents including sodium dihydrogen phosphate, sodium hydroxide, acetic acid and sodium chloride were also obtained from Sigma-Aldrich Co. Soybean oil was purchased from Taiwan Sugar Corp. (Tainan City, Taiwan), while lecithin was obtained from Cheng-Fan Co. (Taipei, Taiwan) and Tween 80 from Yu-Pa Enterprise Co. (Taipei, Taiwan).

Materials for animal experiments including attane (isoflurane), blood collection needle, reusable biopsy punch (8.0 mm), razor, operating knife handle and intravenous injection fluid (30 mg/mL) were all obtained from the Tai-Ho Instrument Co. (New Taipei City, Taiwan), while the blood glucose monitor (GM-700SB) was obtained from Hua-Kuan Biotech Co (Taichung, Taiwan) and blood glucose test paper from I-Kan Instrument Co. (Taipei, Taiwan).

### 2.2. Extraction and Purification of Collagen Peptide from Sturgeon Fish Skin 

A method based on Hou and Chen [2] was used to extract and purify collagen peptide from sturgeon fish skin. Initially, a 100 g sample of dried sturgeon fish skin was collected and soaked in sodium hydroxide (1:10, *w*/*v*) at 4 °C via stirring for 24 h, while the sodium hydroxide solution was replaced every 8 h for removal of non-collagen and fat. Then, distilled water was added to maintain pH at neutral, followed by adding 0.75 M acetic acid (1:10, *w*/*v*), sonication at 4 °C for 30 min (20 kHz, 80% amplitude), extraction at 4 °C for 72 h via stirring and filtration to obtain residue and filtrate. Next, 0.75 M acetic acid was added to the residue (10:1, *v*/*w*) with 0.1% pepsin (400 units/mg), after which the mixture was sonicated at 4 °C for 30 min and then extracted at 4 °C for 72 h via stirring, followed by filtration to obtain filtrate, adding 2.6 M sodium chloride for salting out at 4 °C for 48 h, centrifuging at 18,000× *g* (4 °C) for 30 min, dissolving residue (about 20 g) in 0.1 M acetic acid (40 mL), dialysis (MW cut off 25 kDa) with 0.02 M sodium dihydrogen phosphate for enzyme inactivation, dialysis again with 0.1 M acetic acid and then dialysis again with distilled water. Then, the dialysate (250 mL) was collected and freeze-dried to obtain 5 g of pepsin soluble collagen (PSC) powder for preparation of collagen peptide nanoemulsion with high molecular weight (37 kDa). Next, the PSC sample was collected and mixed with distilled water (1:40, *w*/*v*), after which 5% flavourzyme (≥1.5–10 U/g) was added and this mixture was heated at 50 °C for 4 h for hydrolysis with pH at 8. Then, the collagen peptide was further purified through ultrafiltration with a dialysis membrane with MW cut off at 500 Da for preparation of the collagen peptide nanoemulsion with low molecular weight (728 Da). A total of 250 mL dialysate was collected and freeze-dried to obtain 3 g of collagen peptide powder. Also, the PSC was subjected to SDS-PAGE analysis for MW determination.

### 2.3. Determination of Collagen Variety and Peptide MW Using SDS-PAGE 

A method based on Hou and Chen [2] was used to determine collagen variety and peptide MW using SDS-PAGE. In brief, collagen hydrolysate and PSC were dissolved separately in buffer solutions containing 5% β-mercaptoethanol, 5% mM β-bromophenol, 1.2% Tris-HCl, 10% SDS and 50% glycerol, and mixed with 9.5 mL of deionized water at a ratio of 4:1 (*v*/*v*), followed by heating at 95 °C for 40 min, centrifuging at 12,400 rpm for 10 min, and collecting the supernatant for separation on the stacking and resolving gels under 120 V for 1.5 h. Then, the protein and peptide bands were observed through decolorization with 10% acetic acid and 40% methanol after staining with Coomassie brilliant blue R-250 (0.1%) for 24 h for subsequent determination of collagen and peptide MW through comparison with the protein standards of Sigma marker.

### 2.4. MW Distribution of Peptide via LR-MALDI-TOF/MS

A method based on Hou and Chen [2] was used to determine MW distribution of collagen peptide extracted from sturgeon fish skin using a Bruker Autoflex III Smartbeam MALDI-TOF/MS from the Bruker Corporation (Billerica, MA, USA). Briefly, a sample of collagen peptide supernatant (1 mL) was poured into a dialysis bag in a water bath (4 °C) via stirring for 12 h, followed by mixing with deionized water (10 μL) containing 5% formic acid, desalting using a Zip Tip with C18 resin (0.2 μL), mixing with a sample (1 mL) of 50% acetonitrile, collecting a portion (2 μL) and mixing with 2,5-dihydroxybenzoic acid for deposition onto a target plate, drying for co-crystallization of analyte and matrix, and obtaining mass spectra with linear and positive ion mode.

### 2.5. Amino Acid Analysis of Collagen Peptide via HPLC 

The amino acid composition of collagen peptide was analyzed via HPLC using a method as described by TFDA [10]. Briefly, a 0.5 g sample of collagen peptide was collected and mixed with 20 mL of 0.1 N hydrochloric acid, followed by sonication for 10 min, dilution to 25 mL with 0.1 N hydrochloric acid, filtration through a 0.22 μm membrane filter, collecting filtrate (20 μL) and mixing with 100 μL of boric acid buffer solution (0.4 M). Then, 20 μL of o-phthalaldehyde was added to this mixture for vortex for 60 min, followed by adding 20 μL of 9-fluorenylmethyl chloroformate, vortex for 30 min, adding deionized water (1280 μL) and mixing thoroughly for HPLC analysis. Prior to HPLC analysis, a derivatized amino acid standard solution was prepared via mixing 20 μL with 100 μL of boric acid buffer solution (0.4 M), adding o-phthalaldehyde solution (20 μL), vortex for 60 s, adding 9-fluorenylmethyl solution (20 μL), vortex for 30 s, adding deionized water (1280 μL) and then mixing thoroughly. The various amino acids in collagen peptides were separated using a Poroshell HPH-C18 column (10 cm x 3.0 mm ID, particle size 2.7 μm) and a gradient mobile phase of sodium dihydrogen phosphate (pH 7.8) and acetonitrile/methanol/water (45:45:10, *v*/*v*/*v*) with column temperature at 40 °C, injection volume at 10 μL, flow rate at 0.5 mL/min and detection wavelength at 338 nm and 262 nm. Identification was carried out via comparing retention times and absorption spectra of unknown peaks with reference standards, while quantitation was performed using a formula as described by TFDA [10].

### 2.6. Determination of Degree of Hydrolysis of PSC and Collagen Peptide via O-Phthalaldehyde (OPA) Method 

A method as described by Church et al. [11] was used to determine the degree of hydrolysis of PSC and collagen peptide via a spectrophotometer. Briefly, a 20 μL sample of PSC or collagen peptide solution was mixed with OPA reagent (1 mL) and reacted at room temperature for 2 min for the absorbance measurement at 340 nm. The serine standard curve was also prepared and used as a basis for conversion into degree of hydrolysis using a formula as described by Church et al. [11].

### 2.7. Preparation of Collagen Peptide Nanoemulsion 

A 0.1 g of collagen peptide powder from PSC was collected in a tube, after which 50 mg of distilled water (0.5%) and 0.2 g of soybean oil (2%) were added for complete stirring, followed by adding 0.4 g of lecithin (4%), 0.8 g of Tween 80 (8%) and 8.55 g of deionized water (85.5%) for complete stirring (Table 1). Then, this mixture was shaken in an ultrasonicator for one hour to obtain a 10-mL of collagen peptide (MW 37 kDa) nanoemulsion containing peptide at 10,000 μg/mL. Similarly, for preparation of 10 mL of collagen peptide (MW 728 Da) nanoemulsion containing peptide at 10,000 μg/mL, 0.1 g of collagen peptide powder was collected in a tube and mixed with 100 mg of distilled water (1%) and 0.12 g of soybean oil (1.2%) for complete stirring. Then, 0.05 g of lecithin (0.5%), 0.6 g of Tween 80 (6%) and 9.13 g of deionized water were added for complete stirring, followed by shaking in an ultrasonicator for one hour.

### 2.8. Characteristics Determination of Collagen Peptide Nanoemulsion

The mean particle size and polydispersity index of collagen peptide nanoemulsion were determined via collecting a 100 μL of sample, diluting to 5 mL with 25 mM of dihydrogen potassium phosphate (pH 5.3–5.5) and collecting 3 mL for filtering through a 0.22 μm membrane filter (Nylon, 13 mm) with an injection needle, and with an upper limit of 300–500 kilo counts per second of the dynamic light scattering (DLS) instrument. In addition, the mean particle size and shape of collagen peptide nanoemulsion were determined via a transmission electron microscope (TEM). A sample was collected and diluted 50 times with deionized water, after which a portion (20 μL) was collected and dropped onto a carbon-coated copper grid for 60 s, followed by removing excess sample with a filter paper, negative staining with 20 μL of phosphotungstic acid for 60 s, drying in an oven for 24 h and then enlarging 3 × 10^5^ times under 120 kV for observation via TEM. Also, the zeta potential of collagen peptide nanoemulsion was determined by collecting a 0.1 g sample, diluting it 50 times with deionized water, filtering through a 0.22 μm membrane filter and measuring at 25 °C using a zeta potential analyzer.

### 2.9. Stability Study of Collagen Peptide Nanoemulsion

The collagen peptide nanoemulsion was stored at 4 °C and 25 °C for 3 months, during which a portion was collected at the 15th, 30th, 45th, 60th, 75th and 90th day for determination of mean particle size and PDI via DLS, as well as zeta potential using a zeta potential analyzer. Similarly, for the heating stability study, a 200 μL sample was collected in a tube, which was then placed in a water bath with temperature controlled at 40, 60, 80 and 100 °C for 0.5, 1, 1.5 and 2 h. A total of 48 tubes (triplicate study) were used and all the samples were determined for mean particle size and PDI via DLS as well as zeta potential using a zeta potential analyzer.

### 2.10. Animal Experiment

A total of 96 mice including 88 7-week-old db/db mice (BKS. Cg-Dock7m+/+Leprdb) and 8 7-week-old C57BL/6 male mice were procured from National Experimental Animal Center (Taipei, Taiwan) and then transported to Fu Jen University Experimental Animal Center. This experiment was approved by the Institutional Animal Care and Use Committee, Fu Jen University with an approval number A10940. All the mice were housed in individual ventilation cages with the temperature at 21 ± 2 °C and relative humidity at 55 ± 10% for 12 h under light and under a sterile environment. All the mice were fed with a lab diet 5001 and water ad libitum. After adaption for one week, all the 96 8-week-old mice were ready for experiments and divided into 12 groups with 8 in each:

1. Positive control group (C): a total of 8 C57BL/6 male mice were used for wound opening with a round shape (0.85 cm diameter) and tube feeding with distilled water started at the second day for 14 days.
2. Control group (D): a total of 8 db/db mice were used without wound opening and tube feeding with distilled water every day for 14 days.
3. Induction group (E): a total of 8 db/db mice were used without wound opening and tube feeding with metformin at 250 mg/kg/day for 14 days to induce diabetes.
4. Wound control group (F): a total of 8 db/db mice were used for wound opening with a round shape (0.85 cm diameter) and tube feeding with distilled water started at the second day for 14 days.
5. Low-molecular-weight (728 Da) and low-dose extract group (LLE): a total of 8 db/db mice were used for wound opening with a round shape (0.85 cm diameter) and tube feeding with LLE at 100 mg/kg/day started at the second day for 14 days.
6. Low-molecular-weight (728 Da) and high-dose extract group (LHE): a total of 8 db/db mice were used for wound opening with a round shape (0.85 cm diameter) and tube feeding with LHE at 300 mg/kg/day started at the second day for 14 days.
7. High-molecular-weight (37 kDa) and low-dose extract group (HLE): a total of 8 db/db mice were used for wound opening with a round shape (0.85 cm diameter) and tube feeding with HLE at 100 mg/kg/day started at the second day for 14 days.
8. High-molecular-weight (37 kDa) and high-dose extract group (HHE): a total of 8 db/db mice were used for wound opening with a round shape (0.85 cm diameter) and tube feeding with HHE at 300 mg/kg/day started at the second day for 14 days.
9. Low-molecular-weight (728 Da) and low-dose nanoemulsion group (LLN): a total of 8 db/db mice were used for wound opening with a round shape (0.85 cm diameter) and tube feeding with LLN started at the second day for 14 days.
10. Low-molecular-weight (728 Da) and high-dose nanoemulsion group (LHN): a total of 8 db/db mice were used for wound opening with a round shape (0.85 cm diameter) and tube feeding with LHN started at the second day for 14 days.
11. High-molecular-weight (37 kDa) and low-dose nanoemulsion group (HLN): a total of 8 db/db mice were used for wound opening with a round shape (0.85 cm diameter) and tube feeding with HLN started at the second day for 14 days.
12. High-molecular-weight (37 kDa) and high-dose nanoemulsion group (HHN): a total of 8 db/db mice were used for wound opening with a round shape (0.85 cm diameter) and tube feeding with HHN started at the second day for 14 days.

### 2.11. Wound Opening of Mice

All mice were placed in a sealed transparent box separately for anaesthetization with the inhalation anesthetic isoflurane. After mice became unconscious, they were covered with masks to maintain anaesthetization until the wound opening operation was completed. Following anaesthetization, each mouse was placed on an operation lift covered with a blanket to prevent hypothermia, followed by removing the back hair of mice with a razor, sterilizing with 75% alcohol for the mice back and surgical knife, and opening a wound with a round shape (0.85 cm diameter) and depth into the panniculus adiposus layer using a biopsy punch coupled with a scissor.

### 2.12. Measurement of Wound Area

After wound opening and tube feeding of peptide samples, all mice were placed in a sealed transparent box separately for anaesthetization with the inhalation anesthetic isoflurane on day 1, 3, 6, 8, 10, 12 and 15. After the mice became unconscious, they were covered with masks to record the wound area photographed by a camera. Alternatively, a scab in the wound was removed before photography to prevent the wound area from being miscalculated. The wound picture was then measured for the wound area with an Image J software system (version 1.53t) using the following formula:

$$\mathrm{Remaining}\;\mathrm{wound}\;\mathrm{area}\;(\%)=\frac{\mathrm{remaining}\;\mathrm{wound}\;\mathrm{area}}{\mathrm{original}\;\mathrm{wound}\;\mathrm{area}}\;\times \;100$$

### 2.13. Measurement of Fasting Blood Glucose

After wound opening, all mice were placed in a sealed transparent box separately for anaesthetization with the inhalation anesthetic isoflurane on day 1, 4, 10 and 15. After mice became unconscious, blood was collected from mice cheeks for measuring fasting blood glucose.

### 2.14. Tissue Pathological Section

Following sacrifice, mice organs including liver and kidney were collected and fixed with 10% formaldehyde, while mice wound skin were also collected for clinging to thick cardboard to prevent shrinkage and then fixed in 10% formaldehyde. Next, mice livers, kidneys and wound skin were subjected to rough retouching for subsequent pathological section via staining with hematoxylin and eosin.

### 2.15. Statistical Analysis

All the experiments were conducted in triplicate and data expressed as mean ± SD (standard deviation). Additionally, all the data were subjected to statistical analysis using a statistical analysis system [12] for analysis of variance using one-way ANOVA and Duncan’s multiple range test for significance in mean comparison (*p* < 0.05).

## 3. Results and Discussion

### 3.1. Extraction and Purification of Collagen Peptide from Sturgeon Fish Skin

**Figure 1:**
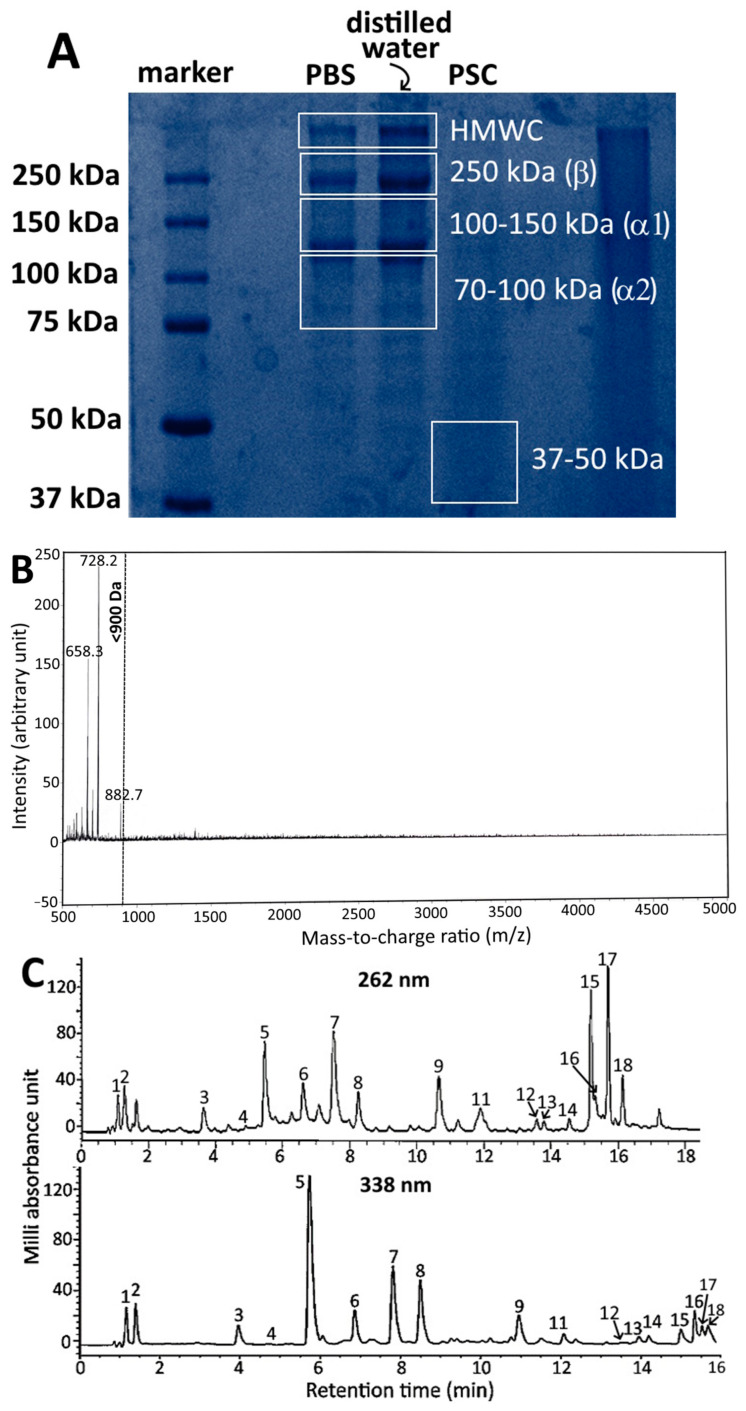
SDS-PAGE of sturgeon fish skin collagen dissolved in two different solvents and PSC obtained via acetic acid followed by 0.1% pepsin and ultrasonication (**A**) LR-MALDI-MS chromatogram of PSC from sturgeon fish skin after treatment with 0.1% pepsin followed by 5% flavourzyme (*w*/*w*) and purification using ultrafiltration membrane with MW cut off at 500 Da; (**B**) HPLC separation of 16 amino acids in sturgeon fish skin extract with ultraviolet detection at 262 nm and 338 nm; (**C**) Peaks: 1, aspartic acid; 2, glutamic acid; 3, serine; 4, histidine; 5, glycine; 6, threonine; 7, arginine; 8, alanine; 9, tyrosine; 10, cystine; 11, valine; 12, methionine; 13, phenylalanine; 14, isoleucine; 15, leucine; 16, lysine; 17, hydroxyproline; 18, proline. SDS-PAGE, sodium dodecyl sulfate polyacrylamide gel electrophoresis; PSC, pepsin soluble collagen; PBS, phosphate-buffered saline; HMWC, high molecular weight collagen; LR-MALDI-MS, low resolution-matrix assisted laser desorption/ionization-mass spectrometry.

Based on a previous study by Hou and Chen [2], who evaluated the various extraction and purification methods for isolation of collagen peptides from sturgeon fish skin, it was shown that with acetic acid (0.75 M) and ultrasonic extraction (30 min), a high yield of sturgeon fish skin collagen (acid soluble collagen, ASC) was obtained. However, following addition of acetic acid (0.75 M) and pepsin (0.1%, 400 units/mg) to the residue obtained during preparation of ASC and for sonication for 30 min, a high yield of pepsin soluble collagen (PSC) was shown. SDS-PAGE analysis revealed the presence of PSC with MW at about 37 kDa through comparison with the protein standards of Sigma Marker (37–250 kDa) (Figure 1A). Furthermore, following addition of 5% flavourzyme (≥500 U/g) to PSC for hydrolysis for 7 h with temperature controlled at 50 °C and pH at 8.0 for subsequent ultramembrane dialysis with MW cut-off at 500 Da, a total of three fragments with MW < 900 Da were obtained with the collagen peptide with MW < 728 Da accounting for 52% of all the fragments based on LR-MALDI-MS analysis (Figure 1B). Also, based on HPLC analysis, a total of 18 amino acids including alanine, arginine, aspartic acid, cystine, glutamic acid, glycine, histidine, isoleucine, lysine, leucine, methionine, phenylalanine, proline, serine, threonine, tyrosine, valine and hydroxyproline were present in sturgeon fish skin collagen peptide, with glycine, proline and hydroxyproline being the most abundant (Figure 1C). The degree of hydrolysis of PSC and collagen peptide as determined via o-phthalaldehyde (OPA) method was shown to be 23.8% and 26.5%, respectively, both of which were slightly higher than that reported by Hou and Chen [2].

### 3.2. Characteristics of Collagen Peptide Nanoemulsion

**Figure 2:**
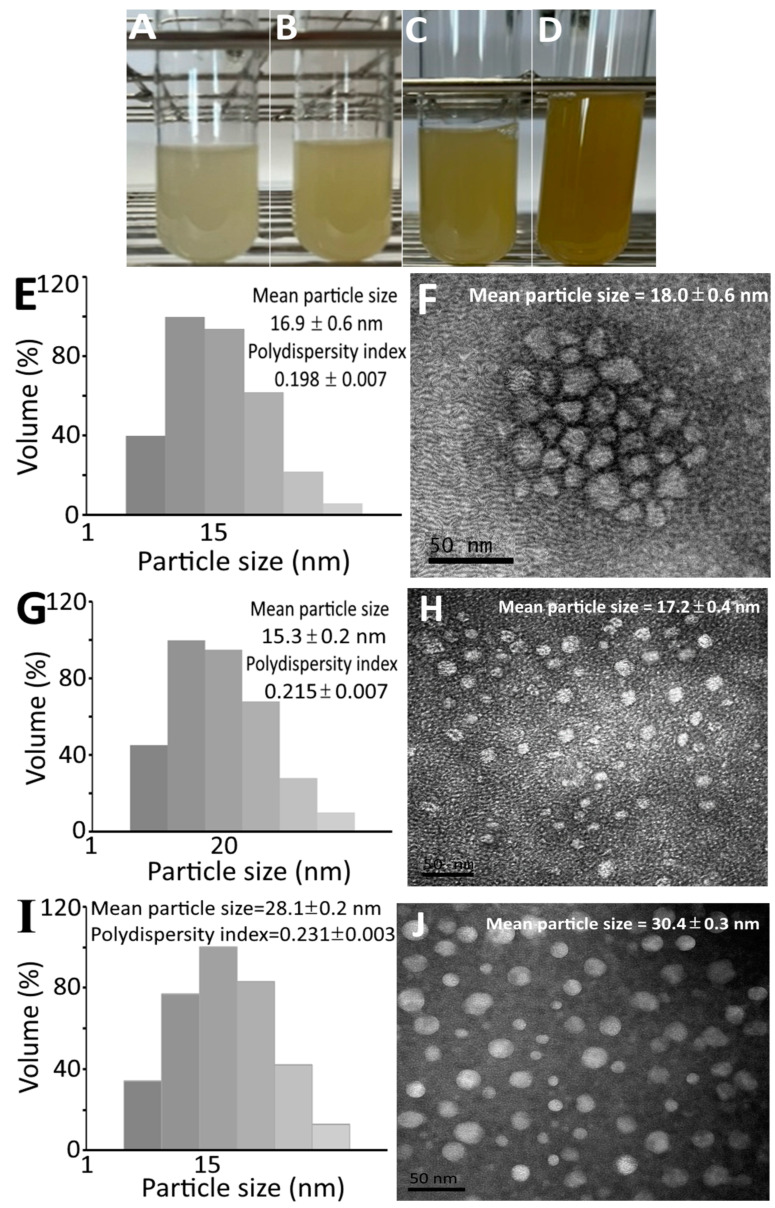

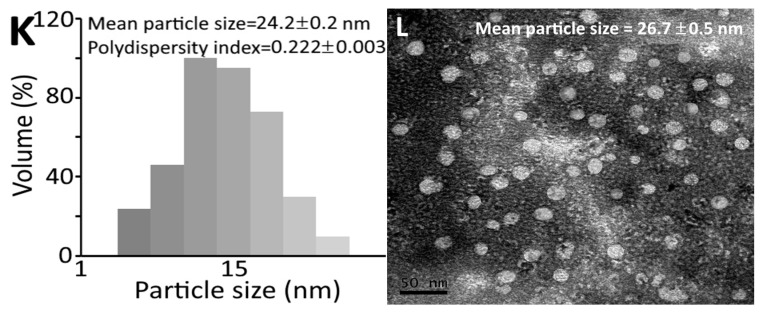
Appearance of collagen peptide nanoemulsions containing peptide at 10,000 μg/mL of low-molecular-weight (728 Da) at low dose (**A**) and high dose (**B**) as well as high-molecular-weight (37 kDa) at low dose (**C**) and high dose (**D**) along with particle size distribution via DLS and surface morphology via TEM of low-molecular-weight and low-dose-nanoemulsion (LLN) (**E**,**F**), low-molecular-weight and high-dose-nanoemulsion (LHN) (**G**,**H**), high-molecular-weight and low-dose-nanoemulsion (HLN) (**I**,**J**) and high-molecular-weight and high-dose-nanoemulsion (HHN) (**K**,**L**). DLS, dynamic light scattering instrument; TEM, transmission electron microscope.

Figure 2 shows the appearance of collagen peptide nanoemulsion containing peptide at 10,000 μg/mL of low molecular weight (728 Da) with low dose (100 mg/kg/day) (Figure 2A) and high dose (300 mg/kg/day) (Figure 2B) and high molecular weight (37 kDa) at low dose (Figure 2C) and high dose (Figure 2D). A transparent green appearance was shown for all the four nanoemulsions, with the color of high-molecular-weight nanoemulsions being deeper than that of low-molecular-weight nanoemulsions. The mean particle size and PDI of LLN as determined via DLS were 16.9 nm and 0.198, respectively, while the mean particle size as determined via TEM was 18.0 nm (Figure 2E,F). Similarly, the mean particle size and PDI of LHN as determined via DLS were 15.3 nm and 0.215, respectively, as well as 17.2 nm based on TEM analysis (Figure 2G,H). However, a larger mean particle size and PDI were shown for HLN, which equaled 28.1 nm and 0.231, respectively, via DLS analysis, as well as 30.4 nm using TEM analysis (Figure 2I,J). Likewise, for HHN, the mean particle size and PDI were 24.2 nm and 0.222, respectively, via DLS, as well as 26.7 nm via TEM (Figure 2K,L). This outcome revealed that the high-molecular-weight peptide nanoemulsion possessed a larger particle size than the low-molecular-weight peptide nanoemulsion. Furthermore, the mean particle size of collagen peptide nanoemulsion prepared from sturgeon fish skin was smaller than that reported by Zhao et al. [13] and Adjonu et al. [14], with the former preparing nanoemulsion from cod bone hydrolysate with the mean particle size being 300–400 nm, and the latter preparing nanoemulsion from whey protein peptide with the mean particle size being 199–290 nm. Apparently, the difference in particle size can be attributed to the variety and amount of ingredients used to prepare the nanoemulsion as well as the concentration and molecular weight of collagen peptide.

DLS is a technique that can be used to determine the size distribution profile of small particles in suspension. Principally, when light strikes small particles, the light scatters in all directions when the particles are small compared to the wavelength (<250 nm). Also, the scattering intensity fluctuates over time if the light source is a laser, caused by the Brownian motion of small particles in suspension. The smaller the particles, the faster the Brownian motion. Thus, the distance between the samples (particles) in the solution is constantly changing with time. Then, the scattered light undergoes constructive or destructive interference via the surrounding particles to obtain the information about the particle size distribution and polydispersity index (PDI) [15].

TEM is a technique which can provide an image at an extremely high resolution, formed through the interaction of a beam of electrons with samples as the beam is transmitted through the specimen with an ultrathin section <100 nm thick. Then, the image is magnified and focused onto an imaging device to obtain the information of both size and shape of nanoparticles [16]. Compared to TEM, DLS is faster, easier to operate and samples can be recovered for reuse. However, DLS can be affected by impurities present in suspension. Conversely, TEM is more specific about the nanoparticle size and shape, but the sample pretreatment is lengthy and samples cannot be recovered for reuse.

### 3.3. Stability of Collagen Peptide Nanoemulsion

The particle size, PDI and zeta potential of collagen peptide nanoemulsion (LLN, 728 Da) during storage at 4 °C and 25 °C for 90 days is shown in Table 2. The mean particle size followed a time-dependent rise over a 90-day storage period at 4 °C and 25 °C, probably caused by partial flocculation after extensive storage. Only a slight change in PDI was shown after 90-day storage, implying a homogeneous dispersion of nanoparticles in the nanoemulsion prepared in our study. For zeta potential, a range from −61.2 to −52.6 mV and −61.2 to −50.8 mV was shown at 4 °C and 25 °C, respectively, over a 90-day storage period, demonstrating a high stability of the collagen peptide nanoemulsion prepared from sturgeon fish skin. It has been well documented that the zeta potential has to be controlled at >30 mV or <−30 mV to maintain high stability of a nanosystem [17]. Similar outcomes were shown for LHN (728 Da) (Table 2), HLN (37 kDa) (Table 3) and HHN (37 kDa) (Table 3) during storage at 4 °C for 90 days, as evidenced by a slight change in particle size from 15.1 to 17.0 nm, 21.9 to 29.2 nm and 16.3 to 24.4 nm, respectively, as well as from 15.3 to 51.6 nm, 28.1 to 50.8 nm and 21.3 to 38.0 nm. This result indicated that a higher storage temperature may induce nanoparticle aggregation. Nevertheless, the mean particle size is still within the nanometer range (<100 nm).

Table 4 shows particle size, PDI and zeta potential changes as affected by LHN (728 Da) during heating at 40, 60, 80 and 100 °C for 0.5, 1.0, 1.5 and 2.0 h. Only a slight change in particle size, zeta potential and PDI was found with a range of 15.0–22.2 nm, −45.4– −32.2 mV and 0.228–0.297, respectively, after heating at 40–100 °C for 0.5–2.0 h. But for HHN (37 kDa), a wider range of particle size (16.5–61.2 nm), zeta potential (−42.2 to −54.0 mV) and PDI (0.219–0.298) was shown over a heating period of 0.5–2.0 h at 40–100 °C (Table 4). This outcome implied that a high heating temperature may also induce nanoparticle aggregation due to Ostwald ripening, a non-homogeneous structural change over time caused by dissolution of small crystals or sol particles and deposition of the dissolved species on the surfaces of large crystals or sol particles. For example, flocculation is often found in oil-in-water emulsions in colloidal systems [18].

However, we have to point out that only LHN (728 Da) and HHN (37 kDa) were conducted for the heating stability study, as both LLN (728 Da) and HLN (37 kDa) should possess similar heating stability. As shown above, all four treatments including LLN (728 Da), LHN (728 Da), HLN (37 kDa) and HHN (37 kDa) exhibited a similar storage ability.

### 3.4. Animal Experiment

Table 5 shows the effect of administration of sturgeon fish skin collagen peptide extract and nanoemulsion on body weight in diabetic and post-traumatic mice. With the exception of C, D and E treatments, all the other treatments followed a time-dependent decline in body weight in diabetic mice with open wounds, as shown by a decrease of 22.37, 14.62, 17.90, 14.63, 20.46, 13.53, 13.80 and 18.50% for LLE, LLN, LHE, LHN, HLE, HLN, HHE and HHN treatments, respectively, which can be attributed to a reduction in appetite caused by feeling pain during healing. Conversely, both D and E treatments only showed a minor change in mice body weight as no wound opening is performed. But for the C treatment (open wound of C57BL/6 mice), only a minor change in body weight was shown, probably due to the presence of a strong immune system. Furthermore, both high-molecular-weight and low-molecular-weight extract and nanoemulsion of a high dose or low dose showed a similar effect in reducing body weight of diabetic mice with open wounds following administration for 15 days. By comparison, the mice body weight of LLE and HLE treatments were reduced to a higher level, indicating that the nanoemulsion may be more efficient than the extract in minimizing body weight loss of diabetic mice with open wounds during 15-day administration.

The effects of administration of sturgeon fish skin peptide extract and nanoemulsion on fasting serum glucose in diabetic and post-traumatic mice is shown in Table 6. With the exception of the positive control treatment, the initial fasting serum glucose levels of the other treatments ranged from 220 to 530 mg/dL, demonstrating the occurrence of type II diabetes in db/db mice. The levels of fasting serum glucose of all the seven treatments including C, D, E, F, LHN, HHE and HHN climbed to the top on the 10th day and then declined thereafter with a minimum being reached on the 15th day after administration. However, a time-dependent increase in fasting serum glucose level was found for LLE, LHE, LLN, HLE and HLN treatments over a 15-day feeding period. Comparatively, the LHN treatment was the most effective in reducing the fasting serum glucose level to a minimum (240 mg/dL) in mice following 15-day administration. It may be postulated that the collagen peptide nanoemulsion with low molecular weight (728 Da) and the high dose should possess higher stability during digestion and absorption in vivo, and thus the β cell functions in the islets of Langerhans were enhanced so that more insulin was secreted to increase the in vivo glucose intake [19].

In addition, the incidence of diabetes can be closely associated with oxidative stress, caused by overproduction of reactive oxygen species (ROS) in vivo. In a study dealing with antioxidative and hypoglycemic effects of tilapia skin collagen peptide in mice, Zhang et al. [20] reported that the fasting blood glucose level was reduced by 31.8% for the peptide treatment and by 30.3% for the metformin treatment. This finding revealed that the high-dose tilapia collagen peptide possessed a similar anti-diabetic effect as the anti-diabetic drug, metformin. Additionally, the tilapia collagen peptide was effective in elevating SOD and CAT activities by 23% and 59.2%, respectively, as well as in scavenging both DPPH and ABTS free radicals [20]. Thus, the tilapia collagen peptide was protective against oxidative damage of pancreatic cells for enhancement of insulin secretion and subsequent reduction in fasting blood glucose level. In another report, Xiong et al. [8] studied the wound-healing effect of the tilapia collagen peptide mixture TY001 in diabetic mice, the high-dose TY001 was the most efficient in reducing fasting blood glucose to a minimum after administration for 15 days.

Moreover, the possible treatment of diabetes through oral delivery of nanoparticles has been well documented. For instance, Sonaje et al. [21] evaluated the safety and efficiency of self-assembled nanoparticles for oral insulin delivery and reported that the fasting blood glucose levels in rats were reduced from 100% to 95%, 78%, 67%, 58%, and 44% after oral intake of insulin encapsulated with nanoparticles for 2, 4, 6, 8 and 10 h, respectively, while the insulin without nanoparticle encapsulation did not show hypoglycemic effect. In a similar study, Li et al. [22] prepared insulin nanoemulsion with alginate and chitosan as coating material as an oral insulin delivery system, the fasting blood glucose level in diabetic rats was reduced by 50% following 6–24 h administration. Specifically, the bioavailability of insulin in diabetic rats was 8.19% and 7.84% at a dose of 25 and 50 IU/kg, respectively. By comparison, the higher bioavailability at a low dose is probably due to higher insulin absorption in blood for subsequent induction of an endogenous adjustment and inhibition of a decline in glucose level through enhanced secretion of glucagon in pancreatic islet α-cells, while a body tolerance may be induced via the excessive exogenous insulin resulting in a decrease in the proportion of insulin absorbed and the hypoglycemic effect [22].

Currently, drugs used for diabetic treatment can only control blood sugar level and the complete cure of diabetes still remains unattainable. Several problems still exist with traditional drug delivery systems for diabetic treatment such as the discomfort of subcutaneous injections of insulin and lack of targeting specificity resulting in adverse side effects of organs/tissues. Moreover, the subcutaneous injection of insulin may cause topical infections, low blood glucose, skin necrosis and nerve damage. To overcome this issue, the oral intake of insulin has been suggested to minimize the adverse side effects, however, the therapeutic effect remains inadequate due to low bioavailability of insulin, a polypeptide with MW 5800 Da composed of 51 amino acids, caused by degradation in stomach/intestine in the presence of gastric juice and enzymes. Thus, the encapsulation of insulin with nanoparticles can not only enhance its aqueous solubility and stability, but also its bioavailability in vivo [23]. The application of using insulin nanoparticles as drug delivery systems has been documented in several previous studies. For instance, Sonaje et al. [21] evaluated the safety and efficiency of self-assembled nanoparticles for oral insulin delivery in diabetic rats; no blood sugar reduction was shown following oral intake of insulin without nanoparticle encapsulation. Conversely, after the oral intake of insulin encapsulated with nanoparticles for 2, 4, 6, 8 and 10 h, the blood glucose levels were reduced by 5, 22, 33, 42 and 56%, respectively. Thus, in our study, the peptide nanoemulsion with low molecular weight (728 Da) may also be used as a drug delivery system for possible treatment of diabetes and wound healing.

### 3.5. Wound Healing Effect in Diabetic Mice

**Figure 3:**
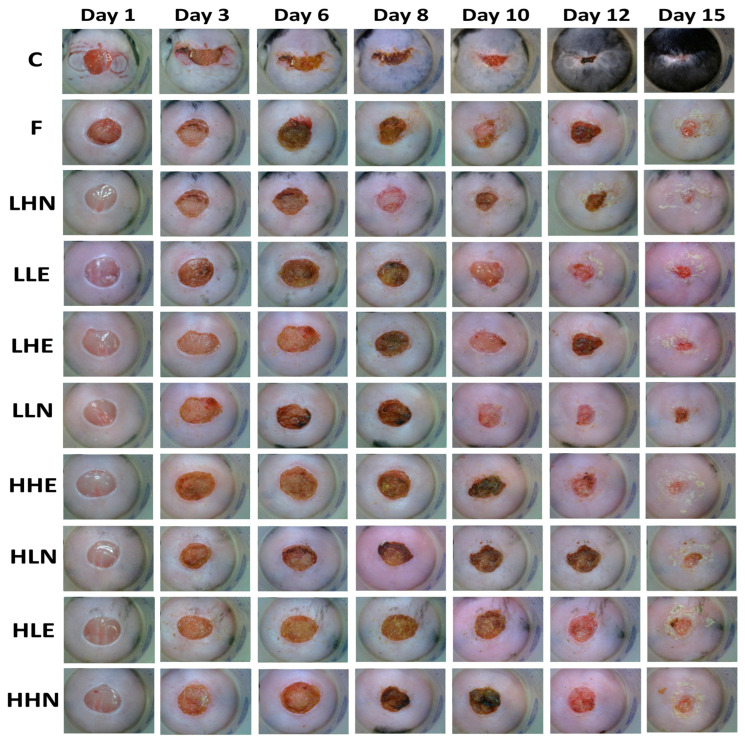
Effects of administration of collagen peptide extract and nanoemulsion on skin excisional area in diabetic and post-traumatic mice. Groups: C, positive control (open wounded C57BL/6 mice fed with drinking water); F, control (open wounded db/db mice fed with drinking water); LHN, low molecular weight and high dose nanoemulsion; LLE, low molecular weight and low dose extract; LHE, low molecular weight and high dose extract; LLN, low molecular weight and low dose nanoemulsion; HHE, high molecular weight and high dose extract; HLN, high molecular weight and low dose nanoemulsion; HLE, high molecular weight and low dose extract; HHN, high molecular weight and high dose nanoemulsion.

The effects of administration of sturgeon fish skin peptide extract and nanoemulsion on skin excisional area in diabetic and post-traumatic mice are shown in Figure 3 and Table 7. The post-wounding area followed a time-dependent decline for all the treatments over a 15-day period, with the positive control treatment (C) showing the smallest post-wounding area (3.09%), followed by LHN (4.47%), LHE (7.77%), HHE (11.68%), LLE (14.04%), F (15.89%), LLN (20.20%), HHN (23.09%), HLN (23.83%) and HLE (25.18%). Like the positive control treatment (96.91%), the LHN treatment resulted in a large total healing area (95.53%), revealing that the low-molecular-weight and high-dose peptide nanoemulsion possessed a pronounced effect in wound healing in diabetic mice. Conversely, the smallest total healing area (74.82%) was shown for HLE treatment, implying that the peptide extract with low dose and high MW was less effective in wound healing in diabetic mice, probably due to lower penetration capability into the skin.

As mentioned above, wound healing usually occurs in four phases (stages) including hemostasis, inflammation, proliferation and remodeling. But for diabetics, a common complication associated with diabetes is peripheral neuropathy leading to foot ulceration. In addition, cardiovascular, cerebrovascular and kidney diseases may occur. These complications lead to the overall slowdown of wound healing.

In the literature, the preparation and application of peptide nanoemulsion in the treatment of diabetes and wound healing has been less explored. However, the possible treatment of wound healing via some other types of nanoemulsion by both in vitro and in vivo models was reported. For instance, an in vitro cell monolayer wound scratch assay showed that the phenytoin-loaded alkyd nanoemulsion prepared from alkyds and Tween 80 resulted in 75–82% scratch closure after 36 h, possessing the potential for promoting topical wound healing through elevated proliferation of epidermal cells [24]. In another in vitro model (ex vivo skin biopsies), Bonferoni et al. [25] prepared α-tocopherol-loaded chitosan oleate nanoemulsion and reported that the cell proliferation on keratinocytes and fibroblast cell cultures was stimulated, indicating the possibility of using this nanoemulsion for topical application in wound healing. For in vivo models, Shanmugapriya et al. [26] prepared a nanoemulsion composed of κ-carrageenan, astaxanthin and α-tocopherol and performed a histopathological study on streptozotocin-induced diabetic mice, this nanoemulsion was shown to restore body weight, reduce fasting blood glucose level, improve glucose tolerance and accelerate wound closure. In another study dealing with the effect of curcumin-encapsulated α-tocopherol nanoemulsion on wound healing in diabetic rats, the analysis of selected skin samples revealed a uniform and even distribution of collagen fibers which enhanced collagen deposition and accelerated the skin tissue regeneration process [27]. All the outcomes shown above demonstrated the possibility of nanoemulsion application in the treatment of diabetes and wound healing in humans.

In addition to nanoemulsion, the effect of bioactive peptides on improving wound healing has been extensively studied. For example, Tang et al. [28] designed a small peptide (tiger 17) containing only 11 amino acid residues (c[WCKPKPKPRCH-NH_2_]) and a strong wound-healing-promoting activity was shown in a murine model of full thickness dermal wound through the induction of macrophages’ recruitment to the wound site, the migration and proliferation of both keratinocytes and fibroblasts and the promotion of a tissue-remodeling phase. The collagen peptides (MW 25 kDa) prepared from the jelly fish *Rhopilema esculentum* were found to enhance re-epithelialization, tissue regeneration and collagen deposition in mice [29]. Similarly, the tilapia collagen peptides’ mixture (TY001) was shown to be effective in promoting wound healing in diabetic mice via increasing growth factors and collagen deposition in the wound, reducing diabetic-induced prolonged inflammation, elevating tissue antioxidants and providing nutritional support [8]. More recently, Hao et al. [30] prepared a novel chitosan/sodium alginate/velvet antler blood peptide hydrogel using an in vitro simulated gastrointestinal digestion and ultrafiltration technique and demonstrated its repair effect on diabetic wounds via regulating angiogenesis, inflammatory response and skin flora, which may be due to the presence of the small peptides in velvet antler blood peptides. In a clinical study, the oral administration of collagen peptides with an average MW of 2.0 kDa to patients with badly healing wounds or postsurgical wounds demonstrated a pronounced effect in wound healing, identifying the efficiency of peptides with small MW in improving wound healing in humans without side effects [31].

In a study dealing with the effect of low MW oligopeptides isolated from sea cucumber on diabetic wound healing in db/db mice, Li et al. [32] reported that following oral administration for 14 days, the oligopeptide group was more effective in wound healing than whey protein group through a substantial reduction in cytokines such as IL-6, IL-8 and TNF-α secreted via macrophages due to inflammation. Similarly, a dose- and time-dependent rise in wound-healing area was shown over a 14-day administration period via tube feeding with low MW peptide (430–1000 Da) prepared from Alaska pollock and peptide (100–1000 Da) from Alaska pollock muscle, with both peptides showing a similar wound-healing rate at high (2 g/kg/bw) or low (0.5 g/kg/bw) doses [7]. In another report, Xiong et al. [8] studied the wound-healing effects of the tilapia collagen peptide mixture TY001 in diabetic mice; the highest wound-healing rate was found for high-dose TY001 (91.34%), followed by median-dose (89.34%), low-dose (87.24%) and whey protein (51.56%), demonstrating a superior wound-healing effect of TY001 compared to whey protein.

Alternatively, recent research has been focused on the development of new and effective wound-care materials, especially the nanosized particles due to their capability of promoting wound healing via facilitating proper movement through the healing phases. Among the various nanoparticles, silver nanoparticles gain the most insight due to their excellent chemical stability, high conductivity, catalytic activity and localized surface plasmon resonance, especially for their efficiency against multi-resistant bacteria and bacteria that produce biofilm, which is commonly found in chronic wounds [9]. Nevertheless, the clinical application of silver nanoparticles in wound healing is limited because of their associated tissue toxicities, as evidenced by the accumulation of silver ions in lysosome, activation of the apoptosis-related gene for cell apoptosis and generation of reactive oxygen species leading to alteration in mitochondrial membrane permeability and DNA damage [9]. To overcome this issue, the encapsulation of silver nanoparticles in collagen hydrogels has shown promising safety and efficiency in human skin fibroblasts and keratinocytes as well as their antimicrobial activity against pathogens [33]. In addition to silver nanoparticles, the application of synthetic polymers such as poly(lactic-co-glycolic acid) (PLGA) has been shown to accelerate the wound-healing effect at the site of infection through release of lactate during metabolism [34]. Additionally, PLGA possesses the ability to release loaded drugs continuously to the wound, and thus the wound-healing effect may be enhanced through encapsulation of peptides in PLGA nanoparticles. In full-thickness excisional wounds of mice, Chereddy et al. [35] demonstrated that the administration of LL37 peptide encapsulated in PLGA nanoparticles promoted wound closure through higher collagen deposition, re-epithelialized and neovascularized composition, with the efficiency being higher than when PLGA or LL37 peptide was administered alone. In a later study, Sun et al. [36] prepared carboxymethyl chitosan (CMCS) nanoparticles (258.7 nm) loaded with peptide OH-CATH30 (CMCS-OH30) from king cobra skin and studied its wound-healing effect in mice via comparing with carboxymethyl chitosan nanoparticle and OH-CATH30 peptide treatments; the highest wound-healing rate was observed for CMCS-OH30 (98.5%), followed by OH30 (96%) and CMCS nanoparticle (90%). All the results shown above demonstrated that through encapsulation of collagen peptides with nanoparticles, the wound-healing effect can be greatly enhanced. Collectively, the conjugation of peptides with biodegradable polymers should be a promising candidate for the future treatment of wound healing in humans.

Pressure ulcers, also known as pressure sores, are harmful to the skin and subcutaneous tissues, mainly caused via prolonged pressure or mixed shear force on the skin that covers bony areas of the body such as heels, ankles, hips and tailbones. Accordingly, people confined to bed or sitting in a chair or wheelchair for prolonged periods of time are most likely to develop this disease as lack of blood flow to the skin can lead to skin cell apoptosis or necrosis for subsequent skin infection and wound generation. However, it was reported that the intake of collagen hydrolysate can improve the wound-healing effect of pressure ulcers [37]. Furthermore, collagen peptide with low MW (1200 Da) rich in proline-hydroxyproline was shown to be more efficient in treating patients with pressure ulcers than with high MW (5000 Da) [38]. At least 12 peptides were reported to be present in human blood after intake of collagen peptide [39]. Of the various peptides, proline-hydroxyproline is present in the highest amount in human blood, accounting for about 50% of the total amount of collagen peptides. Thus, the presence of high levels of proline-hydroxyproline in blood should be mainly responsible for the wound-healing effect of pressure ulcers.

### 3.6. Pathological Analysis of Wounded Skin, Kidney and Liver of Mice

**Figure 4:**
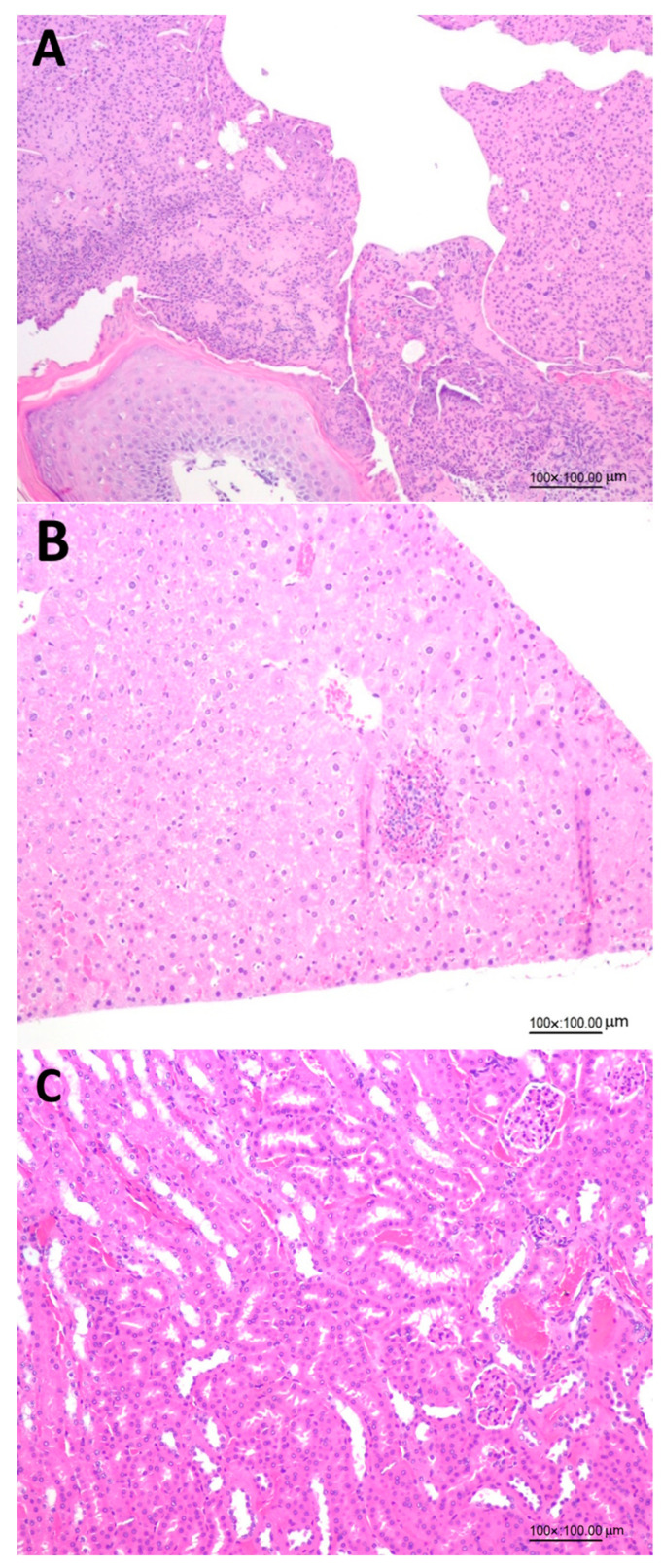
Effects of administration of collagen peptide nanoemulsion (LHN) on histopathology of skin wounds (**A**), livers (**B**) and kidneys (**C**) on day 15 in diabetic mice.

Figure 4 shows the effects of administration of LHN on the histopathology analysis of skin wounds, kidneys and livers in diabetic mice on day 15, demonstrating the safety of the collagen peptide nanoemulsion with low MW prepared in our study. This finding was further confirmed by histopathology of skin wounds (Figure 4A), livers (Figure 4B) and kidneys (Figure 4C) in diabetic mice on day 15 as affected by LHN.

## 4. Conclusions

In conclusion, collagen peptides were prepared from sturgeon fish skin via acetic acid extraction and sonication, followed by one-stage hydrolysis and two-stage hydrolysis with MW of 37 kDa and 728 Da being obtained, respectively. The mean particle size of four nanoemulsions including LLN, LHN, HLN and HHN were, respectively, 16.9, 15.3, 28.1 and 24.2 nm, as well as 0.198, 0.215, 0.231 and 0.222 for PDI and −61.2, −63.0, −41.4 and −42.7 mV for zeta potential. A high storage and heating stability were also shown for these nanoemulsions. Comparatively, the LHN group showed the most pronounced effect in decreasing fasting blood glucose level by 46.75% and maintaining the largest wound-healing area by 95.53% in mice. Thus, the LHN group could be an ideal candidate for development into health foods or wound-healing drugs.

## Figures and Tables

**Table 1:** Ingredients of nanoemulsion formula for high-molecular-weight (37 kDa) and low-molecular-weight (728 Da) peptides.

Ingredient	Percentage (Amount)	
	37 kDa Peptide	728 Da Peptide
Soybean oil	2% (200 mg)	1.2% (120 mg)
Lecithin	4% (400 mg)	0.5% (50 mg)
Tween 80	8% (800 mg)	6.0% (600 mg)
Water	86% (8.6 g)	92.3 (9.23 g)

**Table 2:** Particle size, polydispersity index and zeta potential of sturgeon fish peptide nanoemulsion (LLN and LHN) during storage at 4 °C and 25 °C for 90 days.

Temperature (°C)	Day	Mean Diameter (nm)	Polydispersity Index (PDI)	Zeta Potential (mV)
4		**LLN**		
	0	16.9 ± 0.6 ^A^	0.198 ± 0.007 ^A^	−61.2 ± 0.6 ^A^
	15	23.2 ± 0.2 ^B^	0.250 ± 0.016 ^AB^	−57.2 ± 0.9 ^B^
	30	22.5 ± 0.7 ^B^	0.265 ± 0.003 ^C^	−55.8 ± 0.4 ^C^
	45	24.1 ± 1.0 ^C^	0.220 ± 0.006 ^B^	−53.8 ± 3.3 ^CD^
	60	27.2 ± 0.6 ^D^	0.243 ± 0.005 ^C^	−50.8 ± 0.8 ^E^
	75	30.1 ± 0.6 ^D^	0.298 ± 0.005 ^D^	−52.2 ± 1.9 ^CD^
	90	33.3 ± 0.8 ^E^	0.289 ± 0.005 ^D^	−52.6 ± 1.1 ^CD^
25	0	16.9 ± 0.6 ^A^	0.198 ± 0.007 ^A^	−61.2 ± 0.6 ^A^
	15	36.8 ± 0.3 ^B^	0.299 ± 0.002 ^F^	−44.1 ± 0.5 ^F^
	30	48.2 ± 0.5 ^E^	0.262 ± 0.012 ^D^	−52.3 ± 0.4 ^D^
	45	43.5 ± 0.2 ^C^	0.271 ± 0.008 ^E^	−56.8 ± 0.4 ^C^
	60	44.4 ± 0.5 ^D^	0.254 ± 0.004 ^C^	−61.4 ± 0.5 ^A^
	75	51.2 ± 0.2 ^F^	0.246 ± 0.028 ^B^	−59.6 ± 0.8 ^B^
	90	69.3 ± 0.5 ^G^	0.242 ± 0.008 ^B^	−50.8 ± 0.5 ^E^
4		**LHN**		
	0	15.3 ± 0.2 ^ABC^	0.215 ± 0.007 ^B^	−63.0 ± 0.2 ^A^
	15	15.4 ± 0.2 ^BC^	0.184 ± 0.004 ^A^	−44.3 ± 0.2 ^E^
	30	15.1 ± 0.1 ^A^	0.247 ± 0.005 ^E^	−47.1 ± 0.4 ^C^
	45	15.2 ± 0.3 ^AB^	0.229 ± 0.003 ^C^	−50.7 ± 0.5 ^B^
	60	15.5 ± 0.2 ^C^	0.240 ± 0.012 ^D^	−50.4 ± 0.5 ^B^
	75	16.1 ± 0.2 ^D^	0.255 ± 0.003 ^F^	−47.6 ± 0.4 ^C^
	90	17.0 ± 0.2 ^E^	0.273 ± 0.018 ^G^	−45.1 ± 0.2 ^D^
25	0	15.3 ± 0.2 ^A^	0.215 ± 0.007 ^A^	−63.0 ±0.2 ^A^
	15	38.6 ± 0.1 ^B^	0.304 ± 0.003 ^D^	−44.2 ± 0.9 ^D^
	30	42.6 ± 0.8 ^C^	0.234 ± 0.002 ^C^	−42.0 ± 0.4 ^E^
	45	47.5 ± 0.2 ^D^	0.223 ± 0.002 ^B^	−42.7 ± 1.0 ^E^
	60	49.2 ± 0.3 ^E^	0.235 ± 0.024 ^C^	−43.2 ± 1.0 ^CD^
	75	50.3 ± 0.1 ^F^	0.235 ± 0.003 ^C^	−45.6 ± 1.0 ^B^
	90	51.6 ± 0.6 ^G^	0.229 ± 0.003 ^C^	−43.2 ± 0.3 ^CD^

Data are presented as mean ± standard deviation (n = 3) and values with superscripted capital letters (A–G) are significantly different (*p* < 0.05) in the same column. LLN, low molecular weight (728 Da) and low dose nanoemulsion; LHN, low molecular weight (728 Da) and high dose nanoemulsion.

**Table 3:** Particle size, polydispersity index and zeta potential of sturgeon fish peptide nanoemulsion (HLN and HHN) during storage at 4 °C and 25 °C for 90 days.

Temperature (°C)	Day	Mean Diameter (nm)	Polydispersity Index (PDI)	Zeta Potential (mV)
4		**HLN**		
	0	28.1 ± 0.2 ^C^	0.231± 0.003 ^A^	−41.4 ± 0.7 ^D^
	15	25.2 ± 0.3 ^B^	0.275 ± 0.003 ^C^	−40.4 ± 0.2 ^E^
	30	25.6 ± 0.2 ^B^	0.247 ± 0.004 ^B^	−48.4 ± 0.4 ^C^
	45	29.2 ± 0.3 ^D^	0.233 ± 0.005 ^A^	−51.3 ± 0.5 ^A^
	60	25.4 ± 0.6 ^B^	0.282 ± 0.012 ^D^	−47.8± 0.4 ^C^
	75	21.9 ± 0.6 ^A^	0.340 ± 0.002 ^E^	−49.5 ± 0.3 ^B^
	90	28.4 ± 0.2 ^C^	0.232 ± 0.002 ^A^	−48.6 ± 0.4 ^C^
25	0	28.1 ± 0.2 ^A^	0.231 ± 0.003 ^F^	−41.4 ± 0.7 ^CD^
	15	28.6 ± 0.1 ^B^	0.198 ± 0.005 ^C^	−32.6 ± 0.2 ^E^
	30	50.8 ± 0.3 ^C^	0.282 ± 0.003 ^G^	−40.5 ± 0.4 ^D^
	45	131 ± 0.7 ^E^	0.156 ± 0.026 ^A^	−45.8 ± 0.2 ^B^
	60	129.3 ± 0.4 ^D^	0.203 ± 0.017 ^D^	−50.3 ± 1.6 ^A^
	75	131.6 ± 0.6 ^E^	0.216 ± 0.002 ^E^	−42.1 ± 0.7 ^C^
	90	130 ± 0.7 ^D^	0.183 ± 0.003 ^B^	−47.1 ± 0.8 ^B^
4		**HHN**		
	0	24.2 ± 0.2 ^E^	0.222 ± 0.003 ^A^	−42.7 ± 0.4 ^D^
	15	19.8 ± 0.3 ^C^	0.283 ± 0.004 ^D^	−36.2 ± 0.2 ^E^
	30	17.3 ± 0.3 ^B^	0.295 ± 0.002 ^E^	−42.3 ± 0.3 ^D^
	45	16.3 ± 0.2 ^A^	0.375 ± 0.004 ^F^	−46.5 ± 0.4 ^C^
	60	19.5 ± 0.3 ^C^	0.266 ± 0.002 ^C^	−48.3 ± 0.4 ^B^
	75	21.4 ± 0.2 ^D^	0.246 ± 0.003 ^B^	−50.1 ± 0.2 ^A^
	90	24.4 ± 0.3 ^E^	0.262 ± 0.002 ^C^	−48.1 ± 0.5 ^B^
25	0	24.1 ± 0.2 ^B^	0.222 ± 0.003 ^B^	−42.7 ± 0.4 ^B^
	15	21.3 ± 0.4 ^A^	0.276 ± 0.012 ^D^	−34.9 ± 1.7 ^D^
	30	25.5 ± 0.2 ^C^	0.273 ± 0.004 ^D^	−40.3 ± 0.4 ^C^
	45	32.3 ± 0.3 ^F^	0.224 ± 0.004 ^B^	−45.3 ± 0.4 ^A^
	60	26.5 ± 0.2 ^D^	0.235 ± 0.023 ^C^	−43.2 ± 0.6 ^B^
	75	29.1 ± 0.3 ^E^	0.273 ± 0.002 ^D^	−41.9 ± 0.6 ^B^
	90	38.0 ± 0.2 ^G^	0.215 ± 0.003 ^A^	−42.6 ± 0.3 ^B^

Data are presented as mean ± standard deviation (n = 3) and values with superscripted capital letters (A–G) are significantly different (*p* < 0.05) in the same column. HLN, high molecular weight (37 kDa) and low dose nanoemulsion; LHN, high molecular weight (37 kDa) and high dose nanoemulsion.

**Table 4:** Particle size, polydispersity index and zeta potential changes as affected by sturgeon fish peptide nanoemulsions (LHN and HHN) during heating at various temperatures (40–100 °C) for varied time length (0.5–2 h).

Temperature (°C)	Particle Size (nm)				Zeta Potential (mV)				Polydispersity Index (PDI)			
	0.5 h	1.0 h	1.5 h	2.0 h	0.5 h	1.0 h	1.5 h	2.0 h	0.5 h	1.0 h	1.5 h	2.0 h
					**LHN**							
Control (unheated)	15.2				−45.4				0.277			
40	15.3	15.8	16.2	17.9	−44.2	−43.6	−43.4	−41.9	0.228	0.278	0.292	0.297
60	16.2	17.3	18.2	18.3	−39.8	−42.5	−43.8	−42.5	0.251	0.233	0.271	0.282
80	15.0	16.9	17.8	18.5	−44.5	−43.6	−45.1	−38.4	0.291	0.253	0.242	0.272
100	16.3	17.8	20.8	22.2	−40.3	−36.5	−33.0	−32.2	0.254	0.232	0.257	0.288
					**HHN**							
Control (unheated)	16.5				−54.0				0.263			
40	18.3	19.7	21.8	22.3	−45.0	−46.9	−46.1	−46.2	0.219	0.273	0.283	0.294
60	17.9	20.5	23.3	25.6	−47.6	−46.8	−45.8	−44.2	0.248	0.236	0.240	0.248
80	20.4	22.1	25.7	28.8	−44.9	−43.1	−43.3	−42.2	0.248	0.246	0.262	0.270
100	30.8	49.7	58.1	61.2	−46.5	−45.3	−43.2	−44.5	0.262	0.254	0.270	0.298

LHN, low molecular weight (728 Da) and high dose nanoemulsion; HHN, high molecular weight (37 kDa) and high dose nanoemulsion.

**Table 5:** Effects of administration of sturgeon fish skin peptide extract and nanoemulsion on body weight in diabetic and post-traumatic mice.

Groups	Post-Traumatic Body Weight (g)							
	Before Wounded	Day 1	Day 4	Day 6	Day 8	Day 10	Day 12	Day 15
C	21.69 ± 1.03 ^Fabc^	21.36 ± 1.02 ^Ebc^	20.62 ± 0.96 ^Ed^	22.42 ± 0.89 ^Fab^	22.28 ± 0.93 ^Gab^	21.56 ± 0.97 ^Gcd^	23.41 ± 1.09 ^Ha^	22.55 ± 0.89 ^Fab^
D	36.56 ± 2.91 ^BCDa^	36.18 ± 3.01 ^Bab^	34.26 ± 2.89 ^Bb^	34.98 ± 2.67 ^BCDab^	35.73 ± 2.82 ^Bab^	34.21 ± 2.51 ^Bb^	34.76 ± 2.63 ^Bab^	34.61 ± 2.88 ^Bb^
E	40.39 ± 1.70 ^Aa^	40.01 ± 1.63 ^Aa^	38.64 ± 1.82 ^Aabc^	38.89 ± 1.77 ^Aabc^	39.15 ± 1.88 ^Aab^	37.62 ± 1.93 ^Ac^	37.54 ± 1.91 ^Abc^	37.47 ± 1.94 ^Abc^
F	34.23 ± 2.67 ^DEa^	33.81 ± 2.64 ^CDab^	31.55 ± 2.37 ^Dbc^	32.02 ± 2.56 ^Ec^	32.52 ± 2.67 ^BCabc^	30.33 ± 2.45 ^DEFc^	30.28 ± 2.47 ^EFGc^	29.39 ± 2.78 ^CDc^
LLE	35.22 ± 1.63 ^DEa^	34.86 ± 1.61 ^BCDa^	32.30 ± 1.38 ^CDb^	32.49 ± 1.22 ^CDEb^	31.11 ± 1.00 ^Fc^	30.13 ± 1.09 ^EFc^	30.22 ± 1.45 ^DEFGc^	27.34 ± 1.52 ^Ed^
LHE	36.11 ± 1.93 ^CDa^	35.84 ± 1.97 ^Ba^	33.77 ± 2.10 ^BCbc^	33.50 ± 3.01 ^BCbc^	31.58 ± 3.97 ^Ebc^	31.51 ± 3.36 ^CDEc^	32.42 ± 4.57 ^CDb^	29.63 ± 4.97 ^DEc^
LLN	37.47 ± 1.55 ^Ba^	36.95 ± 1.70 ^Bab^	35.34 ± 1.64 ^Bbc^	35.64 ± 1.76 ^Bbc^	35.71 ± 1.68 ^BCab^	33.32 ± 1.19 ^BCde^	33.54 ± 1.21 ^BCcd^	31.99 ± 1.14 ^Ce^
LHN	33.15 ± 4.34 ^Ea^	32.59 ± 4.35 ^Dab^	30.89 ± 3.78 ^CDcd^	31.60 ± 3.46 ^Ebc^	30.87 ± 2.90 ^Fd^	29.34 ± 2.52 ^Fe^	29.14 ± 2.23 ^Ge^	28.30 ± 1.63 ^DEf^
HLE	38.06 ± 1.26 ^BCa^	37.61 ± 1.18 ^Ba^	35.06 ± 1.98 ^Bb^	35.09 ± 2.20 ^Bb^	34.59 ± 2.31 ^BCb^	32.09 ± 1.75 ^CDc^	31.38 ± 1.86 ^DEFcd^	30.26 ± 2.13 ^CDd^
HHE	35.86 ± 2.58 ^DEa^	35.48 ± 2.83 ^BCa^	33.58 ± 3.00 ^BCDb^	32.81 ± 2.90 ^CDEbc^	32.49 ± 2.99 ^DEcd^	32.01 ± 3.12 ^CDcd^	31.33 ± 3.20 ^DEFde^	30.91 ± 3.43 ^CDe^
HLN	36.50 ± 1.06 ^BCDa^	35.98 ± 1.08 ^Bab^	34.19 ± 1.19 ^BCbc^	33.94 ± 1.10 ^BCDcd^	33.38 ± 1.06 ^CDcd^	32.96 ± 0.96 ^BCde^	32.09 ± 1.28 ^CDEe^	31.36 ± 1.71 ^Ce^
HHN	35.35 ± 1.28 ^BCDa^	35.09 ± 1.24 ^BCa^	32.64 ± 1.29 ^BCDb^	31.97 ± 1.45 ^Ec^	31.68 ± 1.38 ^EFbc^	31.00 ± 1.41 ^Fd^	29.85 ± 1.47 ^FGde^	28.78 ± 1.84 ^DEe^

Data are presented as mean ± standard deviation (n = 8) and values with different superscripted capital letters (A–H) are significantly different in the same column as well as values with different superscripted small letters (a–f) in the same row are significantly different (*p* < 0.05). Groups: C, positive control (open wounded C57BL/6 mice fed with drinking water); D, control (unwounded db/db mice fed with drinking water every day); E, control (unwounded db/db mice fed with metformin (250 mg/kg/day)); F, control (open wounded db/db mice fed with drinking water); LLE, low molecular weight and low dose extract; LHE, low molecular weight and high dose extract; LLN, low molecular weight and low dose nanoemulsion; LHN, low molecular weight and high dose nanoemulsion; HLE, high molecular weight and low dose extract; HHE, high molecular weight and high dose extraction liquid; HLN, high molecular weight and low dose nanoemulsion; HHN, high molecular weight and high dose nanoemulsion.

**Table 6:** Effects of administration of sturgeon fish skin peptide extract and nanoemulsion on serum glucose in diabetic and post-traumatic mice.

Groups	Fasting Serum Glucose (mg/dL)			
	Before Wounding	Post-Wounding		
		Day 4	Day 10	Day 15
C	156.40 ± 22.63 ^Db^	212.40 ± 52.53 ^Fa^	224.20 ± 50.20 ^Ca^	196.40 ± 42.08 ^Bab^
D	459.80 ± 71.65 ^Aa^	487.40 ± 61.90 ^ABa^	527.20 ± 68.50 ^Aa^	505.80 ± 79.00 ^Aa^
E	530.20 ± 70.75 ^Aa^	558.40 ± 48.76 ^Aa^	560.60 ± 0.00 ^Aa^	533.80 ± 16.19 ^Aa^
F	395.00 ± 9.17 ^ABb^	408.00 ± 16.52 ^CDb^	582.66 ± 15.04 ^Aa^	512.33 ± 0.00 ^Aa^
LLE	428.75 ± 42.00 ^Ab^	440.50 ± 90.52 ^BCb^	490.50 ± 25.46 ^Aa^	506.00 ± 19.80 ^Aa^
LHE	415.25 ± 43.47 ^ABb^	414.00 ± 41.09 ^CDb^	385.25 ± 45.80 ^Bb^	496.00 ± 50.77 ^Aa^
LLN	363.25± 68.59 ^ABb^	350.75 ± 78.49 ^DEb^	481.25 ± 52.57 ^Aa^	517.75 ± 75.63 ^Aa^
LHN	451.25 ± 28.99 ^ABa^	357.00 ± 24.75 ^DEc^	382.75 ± 66.47 ^Bb^	240.25 ± 16.97 ^Bd^
HLE	401.66 ± 21.08 ^ABb^	423.00 ± 40.71 ^BCb^	577.00 ± 39.84 ^Aa^	600.00 ± 0.00 ^Aa^
HHE	379.00 ± 44.20 ^Bb^	383.50 ± 44.90 ^CDb^	550.75 ± 39.05 ^Aa^	460.75 ± 82.71 ^Aa^
HLN	275.75 ± 44.99 ^Cb^	249.75 ± 67.93 ^Fb^	511.50 ± 44.74 ^Aa^	521.75 ± 0.00 ^Aa^
HHN	220.00 ± 27.07 ^Cc^	320.00 ± 26.01 ^DEb^	539.00 ± 77.19 ^Aa^	514.00 ± 87.31 ^Aa^

Data are presented as mean ± standard deviation (n = 8) and values with different superscripted capital letters (A–F) are significantly different in the same column as well as values with different superscripted small letters (a–c) in the same row are significantly different (*p* < 0.05). Groups: C, positive control (open wounded C57BL/6 mice fed with drinking water); D, control (unwounded db/db mice fed with drinking water); E, control (unwounded db/db mice fed with metformin (250 mg/kg/day)); F, control (open wounded db/db mice fed with drinking water); LLE, low molecular weight and low dose extract; LHE, low molecular weight and high dose extract; LLN, low molecular weight and low dose nanoemulsion; LHN, low molecular weight and high dose nanoemulsion; HLE, high molecular weight and low dose extract; HHE, high molecular weight and high dose extract; HLN, high molecular weight and low dose nanoemulsion; HHN, high molecular weight and high dose nanoemulsion.

**Table 7:** Effects of administration of sturgeon fish skin peptide extract and nanoemulsion on skin excisional area in diabetic and post-traumatic mice.

Groups	Post-Wounding Area (%)							
	Day 1	Day 3	Day 6	Day 8	Day 10	Day 12	Day 15	Total Healing Area
C	100 ± 5.15 ^Aa^	75.45 ± 3.30 ^Cb^	67.74 ± 3.36 ^Fc^	56.34 ± 4.72 ^Gd^	42.39 ± 3.51 ^DEe^	22.02 ± 1.78 ^EFf^	3.09 ± 1.02 ^Cg^	96.91
F	100 ± 2.31 ^Aa^	88.16 ± 4.91 ^Bb^	85.22 ± 5.22 ^DEbc^	79.72 ± 4.64 ^CDc^	35.40 ± 3.19 ^Fd^	28.25 ± 3.40 ^CDd^	15.89 ± 4.77 ^Be^	84.11
LHN	100 ± 3.00 ^Aa^	98.64 ± 3.38 ^Aab^	94.26 ± 3.23 ^ABb^	54.18 ± 2.91 ^Gc^	42.18 ± 1.92 ^Dd^	25.36 ± 3.63 ^De^	4.47 ± 1.99 ^Cf^	95.53
LLE	100 ± 3.26 ^Aa^	90.82 ± 4.02 ^Bb^	83.49 ± 2.48 ^CDc^	69.60 ± 4.80 ^Ed^	69.07 ± 4.40 ^Bd^	19.82 ± 4.37 ^Fe^	14.04 ± 3.12 ^Bf^	85.96
LHE	100 ± 3.89 ^Aa^	97.47 ± 3.18 ^Aa^	92.34 ± 2.67 ^ABb^	72.3 ± 3.53 ^DEc^	58.85 ± 5.19 ^Cd^	54.91 ± 3.78 ^Ad^	7.77 ± 1.73 ^Ce^	92.23
LLN	100 ± 4.79 ^Aa^	97.77 ± 3.90 ^Aa^	97.02 ± 3.13 ^Aa^	96.95 ± 3.90 ^Aa^	46.33 ± 3.68 ^Db^	26.06 ± 3.58 ^DEc^	20.20 ± 5.98 ^Ac^	79.80
HHE	100 ± 3.48 ^Aa^	85.04 ± 4.08 ^Bb^	82.14 ± 4.35 ^Eb^	65.20 ± 4.57 ^Fc^	39.09 ± 5.31 ^EFd^	18.25 ± 2.58 ^Fe^	11.68 ± 5.73 ^Cf^	88.32
HLN	100 ± 3.09 ^Aa^	97.96 ± 3.88 ^Aa^	92.3 ± 2.06 ^ABb^	80.59 ± 3.51 ^Cc^	76.66 ± 3.29 ^Ac^	50.20 ± 4.03 ^Bd^	23.83 ± 5.32 ^Ae^	76.17
HLE	100 ± 3.50 ^Aa^	96.21 ± 2.59 ^Aa^	95.48 ± 4.94 ^ABab^	89.56 ± 4.72 ^Bb^	66.76 ± 3.27 ^Bc^	40.29 ± 3.02 ^Bd^	25.18 ± 6.45 ^Ae^	74.82
HHN	100 ± 3.54 ^Aa^	99.01 ± 4.25 ^Aa^	95.31 ± 3.95 ^ABa^	75.3 ± 3.79 ^CDb^	57.07 ± 3.37 ^Cc^	31.67 ± 3.15 ^Cd^	23.09 ± 5.40 ^Ae^	76.91

Data are presented as mean ± standard deviation (n = 8) and values with different superscripted capital letters (A–G) are significantly different in the same column as well as values with different superscripted small letters (a–g) in the same row are significantly different (*p* < 0.05). Groups: C, positive control (open wounded C57BL/6 mice fed with drinking water); F, control (open wounded db/db mice fed with drinking water); LHN, low molecular weight and high dose nanoemulsion; LLE, low molecular weight and low dose extract; LHE, low molecular weight and high dose extract; LLN, low molecular weight and low dose nanoemulsion; HHE, high molecular weight and high dose extract; HLN, high molecular weight and low dose nanoemulsion; HLE, high molecular weight and low dose extract; HHN, high molecular weight and high dose nanoemulsion.

## Data Availability

Not applicable.
